# Screening of Genes Related to Sex Determination and Differentiation in Mandarin Fish (*Siniperca chuatsi*)

**DOI:** 10.3390/ijms23147692

**Published:** 2022-07-12

**Authors:** Cheng Yang, Liangming Chen, Rong Huang, Bin Gui, Yangyu Li, Yangyang Li, Yongming Li, Lanjie Liao, Zuoyan Zhu, Yaping Wang

**Affiliations:** 1 State Key Laboratory of Freshwater Ecology and Biotechnology, Institute of Hydrobiology, Chinese Academy of Sciences, Wuhan 430072, China; yangcheng@ihb.ac.cn (C.Y.); liangmingchen@126.com (L.C.); guibin@ihb.ac.cn (B.G.); lyangyu@ihb.ac.cn (Y.L.); lyywh613@ihb.ac.cn (Y.L.); liyongming8080@sohu.com (Y.L.); liaolj@ihb.ac.cn (L.L.); zyzhu@ihb.ac.cn (Z.Z.); wangyp@ihb.ac.cn (Y.W.); 2 University of Chinese Academy of Sciences, Beijing 100049, China; 3 Innovative Academy of Seed Design, Chinese Academy of Sciences, Beijing 100101, China

**Keywords:** *Siniperca chuatsi*, X chromosome, sex determination, sex differentiation, expression difference, structural difference

## Abstract

Mandarin fish has an XX/XY sex-determination system. The female mandarin fish is typically larger than the male. Sex identification and the discovery of genes related to sex determination in mandarin fish have important theoretical significance in the elucidation of the regulation and evolutionary mechanism of animal reproductive development. In this study, the chromosome-level genome of a female mandarin fish was assembled, and we found that LG24 of the genome was an X chromosome. A total of 61 genes on the X chromosome showed sex-biased expression. Only six gonadal genes (LG24G00426, LG24G003280, LG24G003300, LG24G003730, LG24G004200, and LG24G004770) were expressed in the testes, and the expression of the other gene LG24G003870 isoform 1 in the ovaries was significantly higher than that in the testes (*p* < 0.01). Five (except LG24G003280 and LG24G003300) of the seven aforementioned genes were expressed at the embryonic development stage, suggesting their involvement in early sex determination. The expression of LG24G004770 (encoding HS6ST 3-B-like) was also significantly higher in female muscles than in male muscles (*p* < 0.01), indicating other functions related to female growth. ZP3 encoded by LG24G003870 isoform 1 increased the C-terminal transmembrane domain, compared with that encoded by other fish zp3 isoforms, indicating their different functions in sex determination or differentiation. This study provides a foundation for the identification of sex-determining genes in mandarin fish.

## 1. Introduction

Fish can uniquely connect the past and future in vertebrate evolution, and they possess almost all the sex chromosome types in vertebrates. In 2001, a study on >900 tropical freshwater fish reported that 4% of fish had sex chromosome differentiation [1]. At present, there are five main types of sex chromosome determination in fish: (1) XX/XY; (2) ZZ/ZW; (3) XX/XO; (4) ZZ/ZO; (5) complex chromosome [2,3,4].

In the study of fish sex genetics, the identification of sex-specific markers is highly important for the determination of sex chromosomes [3,5,6]. In the early stage, the discovery of sex-specific markers in fish was mostly based on traditional technologies, such as amplified fragment length polymorphism [7], random amplified polymorphic DNA [5,8], and simple sequence repeats [9]. There is no doubt that these methods were effective in the past; however, their disadvantages include difficulties in achieving rapid discovery of sex-specific markers in the whole genome. Therefore, in 2017, our laboratory developed a set of analysis processes for discovering sex-specific markers, successively developed sex-specific markers in *Channa argus* and *Channa maculate*, and conducted high-throughput screening in combination with chelex 100 DNA extraction and high-resolution melting (HRM) scan technology [6,10]. This process, combined with high-throughput screening, provides an efficient and economic technical method for the identification of sex markers in high-value economic fish.

Mandarin fish (*Siniperca chuatsi*) is a rare freshwater cultured fish, belonging to the order Perciformes, family Serranidae, subfamily Sinipercinae [11]. *Siniperca chuatsi* is widely loved by consumers as it is delicious, with no intermuscular bone, and has high nutritional value [12]. It belongs to the XX/XY sex-determination system [13], and the growth rate of the females is 10–20% faster than the males [11]. Sex-biased gene expression is considered to be the basis of the sexual dimorphism phenotype [14], and these genes may be original participants in sex determination and early sex differentiation [15]. The study of sex chromosomes is of great significance for the identification of sex-determining genes and other related basic scientific issues, including the mechanisms associated with sex identification, differentiation, and development [1,2].

Female fish possess a higher homology of sex chromosomes (XX) and better quality for genome assembly than male fish. Therefore, female fish are generally selected for genome sequencing and assembly [16]. In this study, we drew the whole-genome fine map of female *S. chuatsi*. Next, by comparing the next-generation sequencing data of female and male individuals, a sex-specific marker was identified, inferring the X chromosome. Lastly, the expression of genes on the X chromosome was analyzed using gonadal tissues and embryonic development transcriptomes, and the molecular structures of genes with different transcripts were compared. This study provides analytical data for further research on the functional genome of *S. chuatsi*, especially on the regulation of sex determination and differentiation during embryonic development.

## 2. Results

### 2.1. Genome Assembly and Annotation

**Figure 1:**
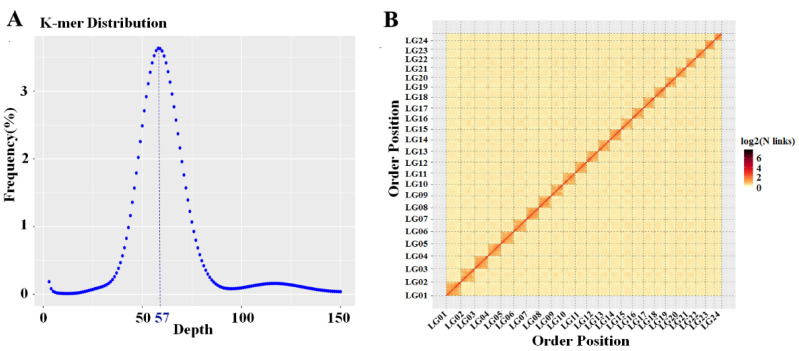
Genome survey and genome-wide Hi-C heatmap of *S. chuatsi*. (**A**) K-mer distribution map of *S. chuatsi* with K = 19; the K-mer depth corresponding to the main peak was 57. (**B**) A genome-wide Hi-C heatmap of *S. chuatsi*; LG 1–24 refer to chromosomes 1–24.

The genome survey results showed that a total of 50.20 gigabases (Gb) of clean data were obtained. Through the K-mers-19 distribution (Figure 1A), we found that the predicted genome size was 734.08 megabases (Mb), the proportion of repeat sequences was 21.11%, the heterozygosity was 0.05%, and the GC content of the genome was 40.36%. After error correction and assembly of Nanopore sequencing data, a draft genome assembly with a total length of 718.79 Mb and contig N50 of 20.48 Mb was obtained. Finally, Hi-C technology was used for chromosome-level assisted genome assembly. The length of the assembled genome scaffolds was 718.80 Mb. A total of 718.52 Mb contigs were mapped to 24 chromosomes. The Hi-C heatmap suggested that the interaction intensity of chromosomes at the diagonal position was higher than at the nondiagonal position, which could clearly distinguish 24 chromosomes, indicating that the genome assembly effect was good (Figure 1B). A total of 23,946 coding genes were predicted in the genome of *S. chuatsi*, and the numbers of tRNAs, rRNAs, miRNAs, and pseudogenes were 1371, 254, 386, and 582, respectively (Supplementary File S1).

### 2.2. Sex-Specific Marker Screening and X Chromosome Identification

**Figure 2:**
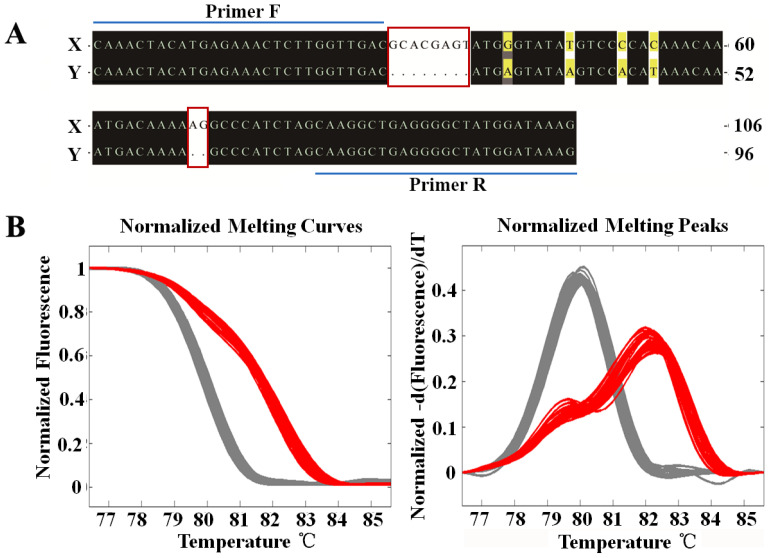
Screening and validation of a sex-specific marker. (**A**) Sequence differences of a sex-specific marker in *S. chuatsi*. Indels are marked with the red boxes; SNPs are marked with a yellow background. The line area indicates the location of upstream and downstream primers. (**B**) The HRM method was used to classify wild females (*n* = 38) and males (*n* = 31). The left and right figures show the normalized melting curves and normalized melting peaks of amplified PCR products, respectively. The gray curve represents females, while the red curve represents males.

Next-generation sequencing (NGS) resequencing was performed on eight females and eight males. After the comparative analysis of female and male reads, the regions with great differences (comparison penalty > 20) between them were obtained, and primers were then designed on both sides of the regions with great differences for polymerase chain reaction (PCR) amplification and HRM typing. The amplification product of a sex marker was 106 bp in females and 96 and 106 bp in males. The difference between the X and Y chromosomes was reflected in two indel loci and four closely linked SNPs loci (Figure 2A). A total of 38 females and 31 males from different water systems were used to verify this marker. The melting curve analysis showed that this marker could effectively distinguish between females and males (Figure 2B). The marker was located in the genome LG24 of the *S. chuatsi* genome, which indicated that LG24 is the X chromosome.

### 2.3. Distribution Characteristics of the Coding Genes and Noncoding Genes on the X Chromosome

In the chromosome-level genome (LG01–LG24) constructed in this study, the sequence from LG01 to LG24 (X) was from long to short. The longest LG01 was 35.71 Mb, and the shortest X was 19.09 Mb. The density distribution map drawn by RIdeogram [17] showed the density distribution of coding and noncoding RNAs on the chromosome of *S. chuatsi* and the position of sex-specific markers on the X chromosome (Supplementary Figure S1). The number of coding genes on the X chromosome was the lowest (587), and that on LG03 was the highest (1266). The X chromosome contained more types of noncoding genes; it had the largest number of tRNAs (124) and rRNAs (140), and the number of pseudogenes ranked second (41). tRNAs and rRNAs constituted two high-density gene transcription units on the X chromosome (Supplementary Figure S1). Pseudogenes were distributed at both ends of each chromosome. The maximum number of miRNAs was 140 on LG11, forming a high-density miRNA cluster, and the predicted miRNAs on other chromosomes (including the X chromosome) did not exceed 30. These findings indicate that the X chromosome was enriched with noncoding genes and comprised a low number of noncoding genes (Supplementary File S1).

### 2.4. Gonadal Differentially Expressed Genes in the HPG Axis and Muscle Tissues

**Figure 3:**
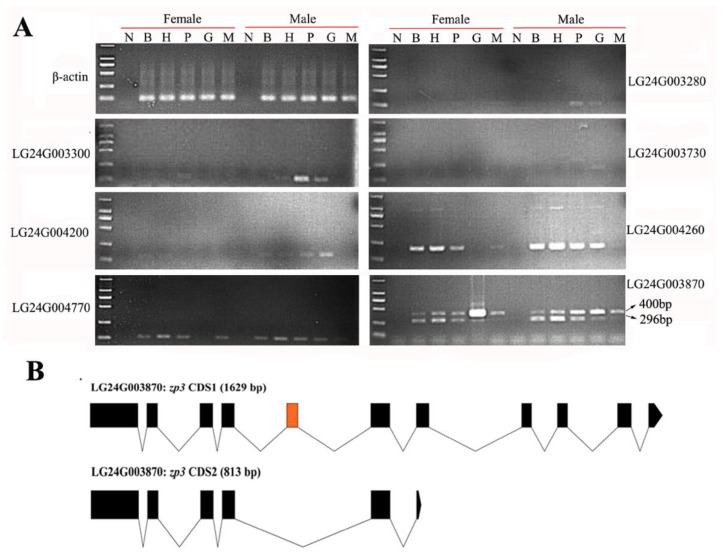
Routine PCR detection of gonadal differentially expressed genes in the HPG axis and muscle tissue and the structural difference of transcripts in one gene. (**A**) The expression of gonadal differentially expressed genes in the HPG axis and muscle tissue was detected by routine PCR. B, H, P, G, and M denote the brain, hypothalamus, pituitary, gonads, and muscle, respectively; N represents the negative control. (**B**) Schematic diagram of the genomic structure of two transcripts of LG24G003870 gene in the CDS region. The orange box indicates the missing exon in CDS2 that causes premature termination of translation.

By analyzing the transcriptome of the testis and ovary of *S. chuatsi*, 61 potential sex-biased genes were identified. We found that the expression of 45 genes in the testes was higher than that in the ovaries, while the expression of the other 16 genes was higher in the ovaries than in the testes (Supplementary File S2). Further routine PCR detection was conducted in the brain, hypothalamus, pituitary, gonads (ovary and testis), and muscle tissues of females and males (Figure 3). The results showed that six genes (LG24G003280, LG24G003300, LG24G003730, LG24G004200, LG24G004260, and LG24G004770) were expressed in the testis but not in the ovary. Furthermore, the expression of the LG24G004770 gene was higher in female muscles than in male muscles, and the LG24G003870 gene could amplify two bands (400 and 296 bp) (Figure 3A). After amplifying the CDS sequences of the two transcripts of the LG24G003870 gene, 400 and 296 bp corresponded to isoforms from two sources: isoform 1 (CDS region length of 1629 bp) and isoform 2 (CDS region length of 813 bp), respectively. Isoform 2 lost the fifth exon of the isoform1 CDS region and terminated the translation in advance (Figure 3B). These seven genes (LG24G003280, LG24G003300, LG24G003730, LG24G003870, LG24G004200, LG24G004260, and LG24G004770) encoded gamma-crystallin M2-like, gamma-crystallin M3-like, carboxypeptidase B2, zona pellucida sperm-binding protein 3 (ZP3), complement C1q-like protein 2, X Kell blood group complex subunit-related family member 6a (XKR6a), and heparan-sulfate 6-*O*-sulfotransferase 3-B-like (HS6ST 3-B-like), respectively.

**Figure 4:**
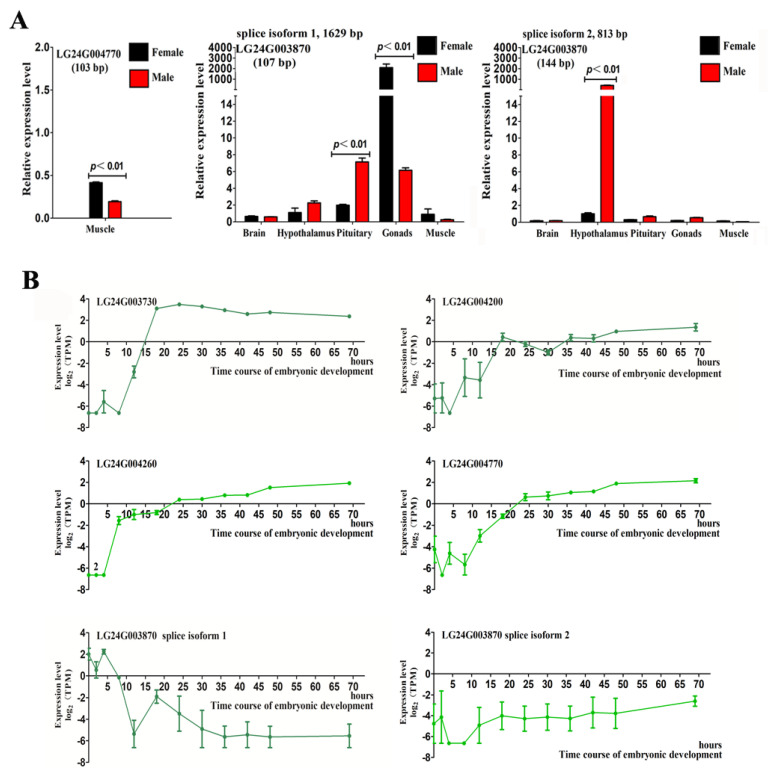
Quantitative polymerase chain reaction (PCR) detection of gonadal differentially expressed genes and transcriptional analysis during embryonic development. (**A**) Quantitative PCR detection of LG24G004770 and LG24G003870 in differentially expressed tissues. The values 103, 107, and 144 bp represent the amplification length. The abscissa represents different tissues, and the ordinate represents the relative expression. (**B**) Transcriptional expression of six transcripts of five genes (LG24G003730, LG24G004200, LG24G004260, LG24G004770, and LG24G003870) in early embryonic development. The abscissa represents the fertilization time, and the ordinate represents the expression level indicated by log_2_ (transcript per million, TPM).

Quantitative PCR detection showed that the expression of the LG24G004770 gene was significantly higher in the female muscles than in the male muscles (*p* < 0.01), and the expression of isoform 1 and isoform 2 of the LG24G003870 gene in the brain, hypothalamus, pituitary, gonads, and muscle was inconsistent (Figure 4A). Isoform 1 was mainly expressed in the gonads, and the expression in the ovary was significantly higher than that in the testis (*p* < 0.01). Isoform 2 was mainly expressed in the hypothalamus, and the expression of isoform 2 was significantly higher in males than in females (*p* < 0.01).

### 2.5. Expression of Gonadal Differentially Expressed Genes during Early Embryonic Development in Mandarin Fish

Transcriptome sequencing was performed on 36 embryo samples at 12 developmental timepoints (Supplementary Figure S2). Overall, 109.78 Gb clean data were obtained after routine filtration. The expression profiles of seven genes were analyzed in the embryonic development transcriptome by determining the transcripts per million (TPM) (Figure 4B). The results showed that two genes, LG24G003280 and LG24G003300, were not expressed during the detected embryonic development period. Moreover, LG24G003730 was gradually upregulated from fertilization to 18 h, maintaining a similar expression level for 18 h. The expression of LG24G004200 was upregulated as a whole, but it fluctuated and reached the highest expression level 69 h after fertilization. LG24G004260 and LG24G004770 exhibited similar expression profile changes and showed upregulated expression in the detection time (0–69 h). The two transcripts isoform 1 and isoform 2 of LG24G003870 had opposing expression patterns, in which isoform 1 was downregulated in the detected embryonic development time and isoform 2 was generally upregulated. The results suggested that two (LG24G003280 and LG24G003300) of the seven genes may be related to sex differentiation or maintenance, and the remaining five genes expressed in the embryo may play a role in sex determination.

### 2.6. Domain Difference Analysis of Two Isomers of LG24G003870 Gene

**Figure 5:**
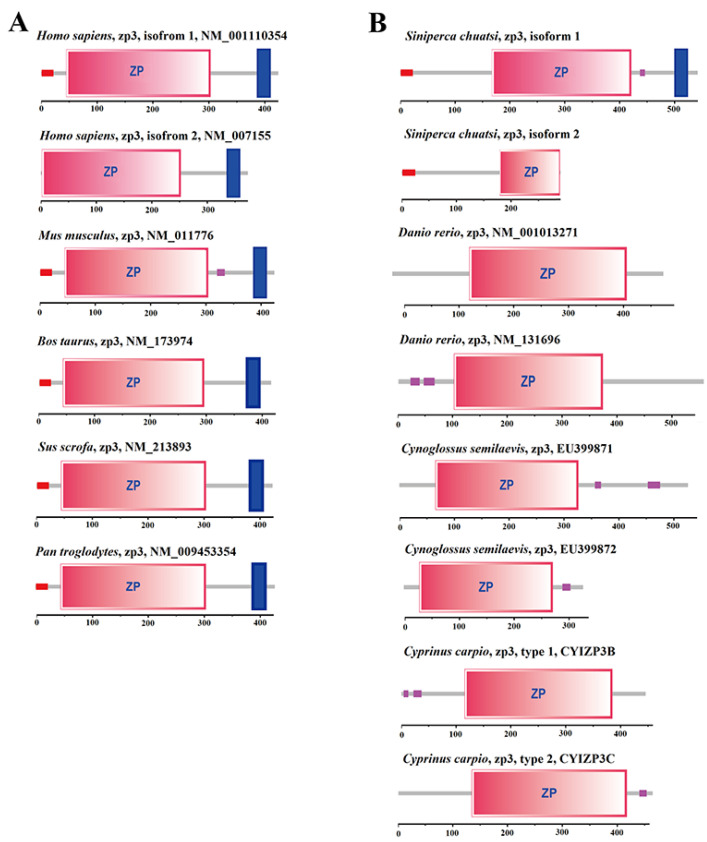
Domain difference analysis of two isomers of the LG24G003870 gene. (**A**) ZP3 protein domains in mammals. (**B**) ZP3 protein domains in fish. The red boxes represent the signal peptide sequence; the pink boxes represent the ZP domain; the blue boxes represent the transmembrane region; the pink and purple boxes represent the low-complexity region.

The protein domains encoded by two transcripts of the LG24G003870 gene of *S. chuatsi* were predicted and analyzed. The results showed that the CDS region of isoform 1 encoded 542 amino acids, including a signal peptide (aa1–aa23), complete zona pellucida (ZP) domain (aa168–aa421), and transmembrane region (aa501–aa523). The CDS region of isoform 2 encoded 270 amino acids and contained a signal peptide (aa1–aa23) and truncated ZP domain (aa168–aa269) (Figure 5). In addition, the ZP3 protein domains of three species of fish (*Danio rerio*, *Cynoglossus semilaevis*, and *Cyprinus carpio*) and five species of mammals (*Homo sapiens*, *Mus musculus*, *Bos taurus*, *Sus scrofa*, and *Pan troglodytes*) were selected for a comparative analysis (Figure 5). Only the ZP3 proteins of *S. chuatsi* contained signal peptides and transmembrane regions similar to those of mammalian species.

## 3. Discussion

Compared with the other chromosomes, the X chromosome in the genome of *S. chuatsi* is the shortest, and it comprises the lowest number of coding genes and almost the highest number of noncoding genes in the genome. In some species, such as flies, worms, and mammals, sex chromosomes have undergone multiple evolutionary processes from a common pair of autosomes [18]. Therefore, compared with autosomal chromosomes, sex chromosomes often have larger differences, which are reflected in the length of sex chromosomes, gene distribution and expression, and mutation rate of chromosomes and genes [19,20]. The X chromosome is longer than the Y chromosome, and the number of genes distributed on it is greater [21]. This suggests that, in the process of evolution, the X chromosome, as a sex chromosome, may have experienced a similar evolutionary selection process to that of the other chromosomes of *S. chuatsi*.

*Siniperca chuatsi* is a gonochoristic fish with a sex determination type of XX/XY and a sex ratio of male to female offspring of approximately 1:1 [22]. This study focused on the sex-determining genes of *S. chuatsi*. Previous studies have reported that there are three main sex determination modes in gonochoristic fish: chromosome determination, polygenic determination, and genotype–environment codetermination [23]. In chromosomal determination, the ratio of male to female is approximately 1:1, and most sex-related genes are concentrated on a pair of sex chromosomes [24]. Therefore, we speculated that sex determination in *S. chuatsi* is chromosomal, and the genes related to sex determination and differentiation are located on its X and Y chromosomes.

Sex determination is an important event in early embryonic development. The expression of genes related to sex determination and differentiation begins during early embryonic development [25]. According to the genome assembly sequence of female *S. chuatsi*, the X chromosome was located using a sex-specific marker, and the expression of genes distributed on the X chromosome in the testis, ovary, and during embryonic development were analyzed. Finally, we observed that the six gonadal genes were only expressed in the testes, and four were expressed during embryonic development. This finding indicated that these four genes (LG24G003730, LG24G004200, LG24G004260, and LG24G004770) are likely to be related to sex determination in *S. chuatsi*. It should be noted that HS6ST 3-B-like was not only expressed in the male gonads but also significantly higher in female muscle than in male muscle (*p* < 0.01). In the early development of zebrafish, HS6ST is enriched in the brain, which is highly important for muscle development. The injection of morpholino targeting HS6ST caused the interruption of muscle development and serious muscle degeneration in zebrafish [26]. The present study speculated that HS6ST 3-B-like of *S. chuatsi* is not only related to sex determination and differentiation, but also participates in the sex dimorphic development process of muscle growth and plays an important role in female growth.

Zona pellucida is an extracellular glycoprotein matrix surrounding the periphery of the egg, which binds to sperm and initiates an acrosome reaction [27]. This study found that the LG24G003870 gene of *S. chuatsi* had two transcripts, isoform 1 and isoform 2, corresponding to the zp3 genes of other species. Prior studies have shown that the zp3 gene contains a variety of isoforms [28,29], and ZP proteins identified in teleosts lack a C-terminal transmembrane region, which is a common feature of mammalian ZP proteins [30]. In the present study, unlike isoform 1, ZP3 encoded by isoform 2 did not contain a transmembrane region. In different teleosts, the zp3 gene is mainly expressed in the ovary or liver [31,32,33]. Although the isoform 1 and isoform 2 of the zp3 gene of *S. chuatsi* were expressed in both female and male fish, the expression levels were not consistent in the different tissues. Isoform 1 was mainly expressed in the gonads, and its expression in the ovaries was significantly higher than that in the testes (*p* < 0.01). Therefore, we speculated that the function of ZP3 encoded by the zp3 gene isoform 1 of *S. chuatsi* is consistent with that of mammals and plays an important role in the process of fertilization. The function of zp3 gene isoform 1 in sex determination and differentiation requires further knockout verification.

## 4. Materials and Methods

### 4.1. Source of Tissue and Embryo Samples of S. chuats

*S. chuatsi* used in the experiment was obtained from the Guanqiao Experimental Fish Breeding Base, Institute of Hydrobiology, Chinese Academy of Sciences. The muscle tissue of a female *S. chuatsi* (F1) was frozen in liquid nitrogen. The DNA of muscle tissue was extracted using the cetyltrimethylammonium bromide method. The quality and concentration of extracted DNA were detected by 1% agarose gel electrophoresis and qubit 3.0 (Thermo Fisher Scientific, Inc., Waltham, MA, USA). Next, genome sequencing and assembly were carried out.

Forty-six sexually mature females (F2–47) and 39 males (M1–39) were randomly selected. The caudal fins were removed, and the DNA was extracted. The caudal fin DNA of eight females (F2–9) and eight males (M1–8) was used for the screening of sex markers. The caudal fin DNA of 38 females (F10–47) and 31 males (M9–39) was used to verify the sex markers. The brain, hypothalamus, pituitary, gonad, and muscle tissues of three females (F3–5) and three males (M2–4) were removed. After grinding with Trizol reagent (Life Technologies, Carlsbad, CA, USA), the same tissues of the same sex were mixed in equal quantities and frozen at −80 °C for gene expression analysis.

F2 and M1 were artificially inseminated, and the fertilized eggs were incubated in aerated water at 25 °C. The unfertilized eggs of F2 were removed once. Next, fertilized eggs were retrieved at 2, 4, 8, 12, 18, 24, 30, 36, 42, 48, and 69 h after insemination (Supplementary Figure S2). Three duplicate samples were obtained at each timepoint. Each duplicate sample contained approximately 100 embryos or 30 fries, which were frozen in liquid nitrogen for transcriptome sequencing. All the schemes of this study were approved by the Ethics Committee of the Institute of Hydrobiology, Chinese Academy of Sciences (approval No.: Y911306).

### 4.2. Assembly and Annotation of S. chuatsi Genome

The 350 bp library was constructed from the muscle tissue DNA of F1. Using NGS by the Illumina platform, 150 bp of paired-end (PE150) reads were generated with an insert size of approximately 350 bp. The clean data were obtained through routine filtration, and K-mers-19 was generated. The genome size, heterozygosity, GC content, and repeat sequence ratio were calculated to evaluate the genome complexity of *S. chuatsi*. Next, the Oxford nanopore long read library was constructed and sequenced on the Nanopore sequencing platform. Canu v1.5 [34] was used for error correction in the clean data; WTDBG v1.2.8 [35] was used for assembly; Racon v1.08 [36] and Pilon v1.23 [37] were used for further correction and to obtain the draft genome assembly. Finally, the Hi-C library was constructed by using the muscle tissue frozen in liquid nitrogen, followed by sequencing with PE150. After routine filtration, valid interaction pairs were obtained using HiC-Pro v2.8.1 [38]. On the basis of valid interaction pairs, the draft genome assembly was further assembled using LACHESIS [39] to obtain the chromosome-level genome assembly. The chromosome-level genome was cut into 100 kb bins of equal length; the number of Hi-C read pairs covering any two bins was used as the signal of the interaction between the two bins, and a heatmap was drawn to evaluate assembly quality.

Genscan [40], Augustus v2.4 [41], GlimmerHMM v3.0.4 [42], GeneID v1.4 [43], and SNAP v2006-07-28 [44] were used for de novo prediction of coding genes. GeMoMa v1.3.1 [45,46] was used for predictions based on homologous species. HISAT v2.0.4 [47] and Stringtie v1.2.3 [48] were used to assemble transcripts with reference sequences, and TransDecoder v2.0 [49] and GeneMarkS-T v5.1 [50] were used to perform gene prediction. PASA v2.0.2 [51] was used to predict unigene sequences on the basis of transcriptome data without reference sequences. EVM v1.1.1 [52] was used to integrate the prediction results obtained from the above methods, followed by modification with PASA v2.0.2. Using the Rfamdatabase [53], BLASTN was employed to perform genome-wide alignment to identify microRNAs (miRNAs) and ribosomal RNAs (rRNAs). Transfer RNAs (tRNAs) were identified using tRNAscan-SE v2.0 [54]. For the prediction of pseudogenes, first, the predicted protein sequence was used to identify the homologous gene sequence on the genome through BLAT [55]; next, GeneWise [56] was employed to find the immature termination codon and a frameshift mutation in the gene sequence to obtain the pseudogene.

### 4.3. X Chromosome Identification and Gene Distribution

A 350 bp library was constructed using the caudal fin DNA of F2–9 and M1–8, followed by NGS sequencing (PE 150). Through a set of biological information analyses, a process developed by our laboratory [6,10], a sex marker of *S. chuatsi* was discovered, and primers (Supplementary File S3) were designed for this marker. After PCR amplification, the HRM system was used to verify the typing of PCR products in F10–47 and M9–39 with the known sex. The process involved placing the PCR plate containing PCR products into the HRM instrument plate slot, followed by scanning at 68–94 °C. The dissolution curves of female- and male-specific sequences were drawn to distinguish male and female individuals. BLAST v2 2.26 was used to match the sex marker with the reference genome, and the matched chromosome was judged as the X chromosome. The distributions of all coding RNAs and noncoding RNAs (miRNA, rRNA, tRNA, and pseudogenes) obtained from genome annotation on the chromosomes of *S. chuatsi* were determined, and the density distribution map was drawn using the R package RIdeogram [17].

### 4.4. Differential Expression Analysis of X Chromosome Gene in the Testis and Ovary

The transcriptome data of three sexually mature testes (SRR11743001) and three ovaries (SRR11743000) of *S. chuatsi* were downloaded from NCBI [57]. After routine filtering, STAR [58] was used to match the transcriptome data with the genome of *S. chuatsi* constructed in this study (Supplementary File S4). Next, the results were analyzed quantitatively using RSEM [59], and the TPM values of all the coding genes on the X chromosome and the read count contained in each gene were obtained. Genes with reads count >5 and a TPM value of 0 in one tissue and not 0 in the other tissue were screened as candidate genes related to sex determination or differentiation.

### 4.5. Expression of Differentially Expressed Genes in the Gonad in HPG Axis and Muscle Tissues

The total RNA of the brain, hypothalamus, pituitary, gonad, and muscle tissues was extracted with Trizol reagent for F2–4 and M1–3. Next, RQ1 RNase-Free DNase (Promega, Madison, WI, USA) was used for DNA digestion. Finally, OligodT (20) (Takara, Kusatsu, Japan) and random primers (Takara, Kusatsu, Japan) were used to reverse-transcribe the total RNA into cDNA under the action of ReverTra Ace (TOYOBO, Osaka, Japan).

The differentially expressed genes in the gonads were amplified by common PCR and then qualitatively analyzed using gel electrophoresis. For genes with multiple bands (possibly from different transcripts), quantitative PCR amplification of different transcripts was carried out after amplifying the sequences of the different transcripts, and the primers were designed in the specific region of transcripts. In addition, the genes differentially expressed in the muscles were amplified by quantitative PCR. Quantitative PCR amplification of each sample was repeated three times, and β-actin was used as the internal reference gene. The relative expression was calculated using the 2^-ΔΔCt^ method and expressed as the mean ± standard deviation. SPSS v25.0 was used for data processing, and the significant differences in the data were evaluated using Student’s *t*-test. The primer sequences are listed in Supplementary File S3.

### 4.6. Expression Analysis of Differentially Expressed Genes in the Gonad during Early Embryonic Development and Structural Analysis of Different Transcripts

The transcriptome of 36 embryo samples (12 timepoints, with three replicates at each timepoint) was sequenced on the basis of NGS (PE150), and clean data were obtained after routine filtration. HISAT2 v2.0.4 [47] was used to align clean data with the reference genome of *S. chuatsi* to obtain mapped data. Mapped reads were used for gene expression analysis (using the TPM calculation method). The proteins encoded by different transcripts and the corresponding transcripts of other species were compared and analyzed. The domain analysis was conducted through SMART (http://smart.embl-heidelberg.de/, accessed on 12 November 2021).

## Data Availability

Raw sequences for genome assembly including Illumina, Nanopore, Hi-C reads, and the transcriptome data related to embryonic development of *S. chuatsi* are available in NCBI (accession number: PRJNA738969). The sequences of LG24G004260, LG24G003280, LG24G003300, LG24G003730, LG24G004200, LG24G004770, LG24G003870 isoform 1, and LG24G003870 isoform 2 were deposited in the GenBank database (accession numbers: ON759753, ON759754, ON759755, ON759756, ON759757, ON759758, ON759759, and ON759760).

## Supplementary Materials

The following supporting information can be downloaded at https://www.mdpi.com/article/10.3390/ijms23147692/s1.
