# Supplementary material for: Screening of Genes Related to Sex Determination and Differentiation in Mandarin Fish (Siniperca chuatsi)
Source: Int J Mol Sci. 2022 Jul 12;23(14):7692. doi: 10.3390/ijms23147692 (PMC9321114; doi:10.3390/ijms23147692)
Supplement: Supplementary file 1 [file ijms-23-07692-s001.zip › ijms-1774323 Supplementary Figures.pdf]

Supplementary Materials

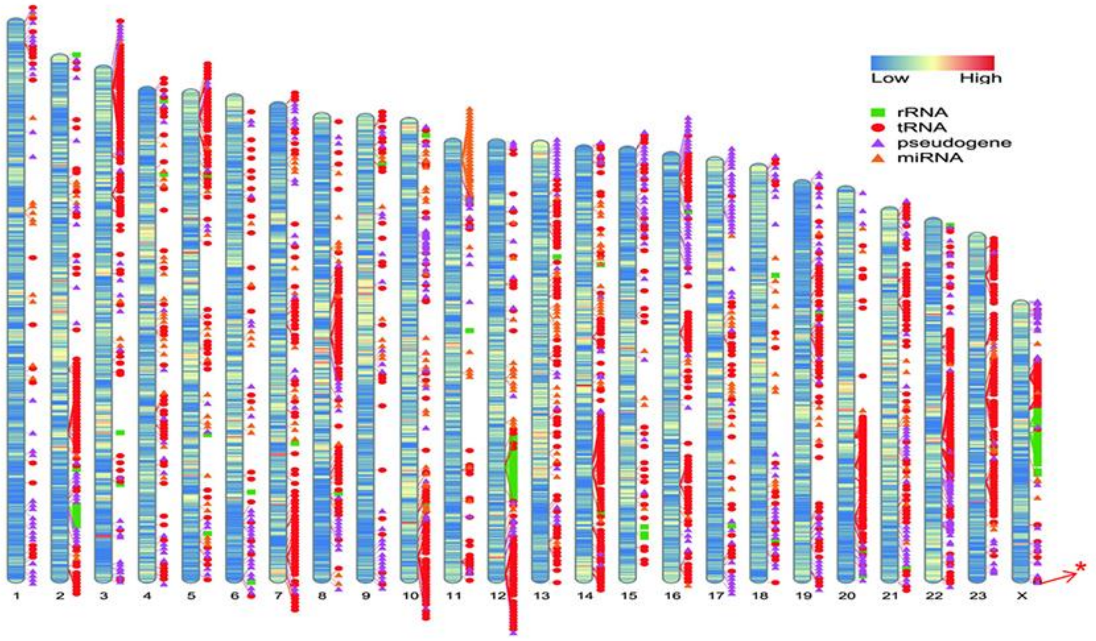

Figure S1. Density distribution of coding genes, rRNAs, tRNAs, miRNAs, and pseudogenes on 24 chromosomes of *S. chuatsi*. Different colors on chromosomes indicate the distribution density of coding genes. The position of the sex marker on the X chromosome is highlighted with a "*" and red arrows.

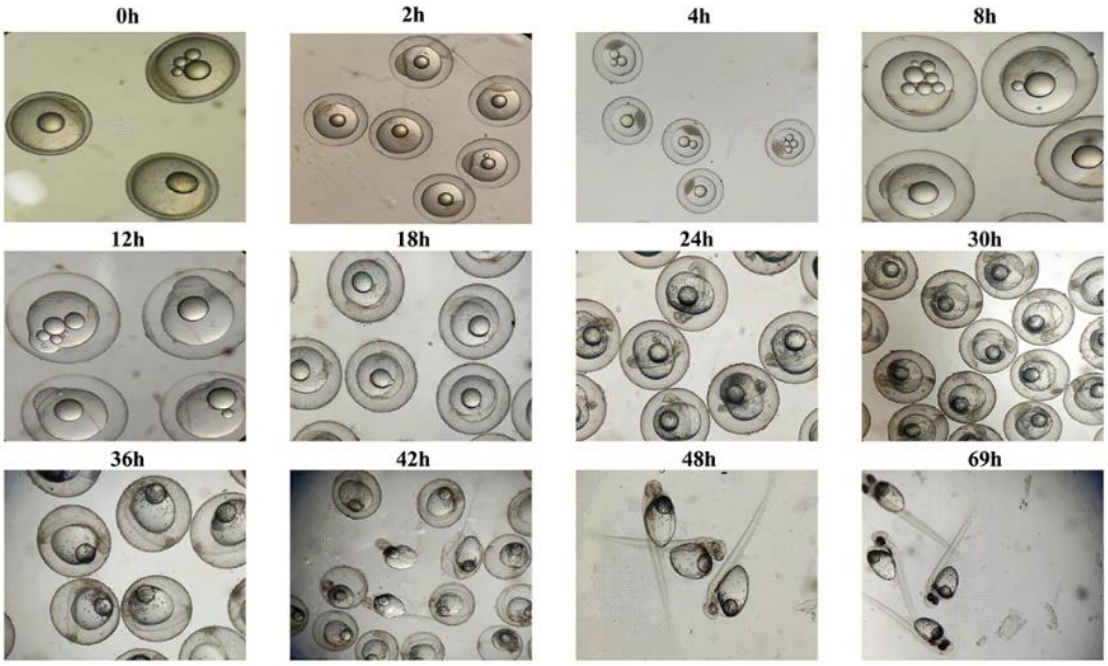

Figure S2. Embryonic morphology of *S. chuatsi* at 12 developmental time points. Each time point and the corresponding embryonic development state are as follows: 0 h-oocyte; 2 h-blastocyst stage; 4 h-globular embryo; 8 h-early gastritis; 12 h-late gastritis; 18 h-tail bud stage; 24 h-embryonic motility; 30 h-heartbeat period; 36 h-early stage of film emergence; 42 h-metaphase of membrane emergence; 48 h-late stage of film emergence; 69 h-eye pigment formation stage.
